# Retinoblastoma in Sub-Saharan Africa: Case Studies of the Republic of Côte d’Ivoire and the Democratic Republic of the Congo

**DOI:** 10.1200/JGO.17.00056

**Published:** 2018-02-07

**Authors:** Robert M. Lukamba, Jean-Jacques A. Yao, Theophile A. Kabesha, Aleine N. Budiongo, Ben B. Monga, Albert T. Mwembo, Pierre Bey, Gabrielle B. Chenge, Laurence Desjardins, Oscar N. Luboya, François Doz, Cristina D. Stefan

**Affiliations:** **Robert M. Lukamba** and **Oscar N. Luboya**, University Clinics of Lubumbashi; **Robert M. Lukamba**, **Ben B. Monga**, **Albert T. Mwembo**, **Gabrielle B. Chenge**, **Oscar N. Luboya**, University of Lubumbashi, Lubumbashi; **Theophile A. Kabesha**, Official University of Bukavu, Bukavu; **Aleine N. Budiongo**, University Hospital of Kinshasa, University of Kinshasa, Kinshasa, Democratic Republic of Congo; **Robert M. Lukamba**, **Pierre Bey**, **Gabrielle B. Chenge**, and **Laurence Desjardins**, Alliance Mondale Contre le Cancer; **Pierre Bey**, **Laurence Desjardins**, and **François Doz**, Institut Curie; **François Doz**, University Paris Descartes, Paris; **Pierre Bey**, University of Lorraine, Lorraine; **Cristina D. Stefan**, International Prevention Research Institute, Lyon, France; and **Jean-Jacques A. Yao**, Centre Hospitalier Universitaire de Treichville, Abidjan, Côte d’Ivoire.

## Abstract

**Purpose:**

In most low-income countries, the diagnosis of retinoblastoma is delayed, resulting in a severe prognosis. The objectives of this study were to describe the access to diagnosis and care of children diagnosed with retinoblastoma and the challenges in two sub-Saharan African countries: the Republic of Côte d’Ivoire and the Democratic Republic of the Congo.

**Patients and Methods:**

A descriptive cross-sectional study was conducted. Data were collected from the medical records of patients admitted during the period of January 1, 2013 to December 31, 2014. Data were entered and analyzed using Epi Info7.1 software and SAS 9.3.

**Results:**

One hundred sixteen cases of retinoblastoma were collected, including 60 boys and 56 girls. The median diagnosis age was 3 years for both countries. Ninety-eight patients (84%) had unilateral retinoblastoma. Most of the patients presented with advanced disease (76% had extraocular retinoblastoma). Median time between initial symptoms and diagnosis was 8.5 months (range, 0.4 to 116.7 months). Median time between diagnosis and treatment initiation was 31 days (range, 0 to 751 days). The median cost for the treatment of the disease was estimated at $1,954 per patient.

**Conclusion:**

Late diagnosis of retinoblastoma, with extraocular disease, occurs frequently in both African countries. It is associated with delay in initiating treatment, and the cost of the treatment remains unaffordable for most of the families. Support groups for parents of affected children and the support of the Franco-African Pediatric Oncology Group remain important in improving early diagnosis and providing treatment in sub-Saharan African countries.

## INTRODUCTION

Retinoblastoma, also known as the cancer of the retina, is one of the most common cancers occurring in young children in sub-Saharan Africa.^1^ The incidence reported in the literature is 9,000 new cases per year, which corresponds to one in 15,000 births.^2^ In developed countries, the median age at diagnosis is ≤ 2 years for unilateral cases (approximately 60% of cases) and ≤ 1 year for bilateral cases.^3,4^

Retinoblastoma is treatable in > 95% of children in developed countries, in which advanced stages have become increasingly rare.^3,4^ The most common symptoms at diagnosis are leukocoria and strabismus. Diagnosis may be performed by screening children at risk with a family history of retinoblastoma and confirmed by fundoscopy under general anesthesia supplemented by ocular ultrasound. In high-income countries, magnetic resonance imaging is performed primarily to assess for extraocular extension.^3,5^

Compared with children in developing countries, the diagnosis of retinoblastoma in children in low-income countries is often delayed, and the prognosis is poor.^6-10^ In most of these low-income countries, mortality from retinoblastoma has reached rates of 40% to 70%.^3^ Sub-Saharan Africa is particularly characterized by a high rate of extraocular forms of retinoblastoma and a poor prognosis.^11-17^

It is known that the delayed diagnosis of retinoblastoma is associated with extraocular forms of the disease and reduced rates of patient survival.^4,18-20^ Several studies have previously described the access to diagnosis and care of retinoblastoma diagnosis and management,^14,21-24^ but few studies have evaluated the factors contributing to delayed diagnosis, particularly in French-speaking sub-Saharan African countries.

Our study was conducted in the Democratic Republic of Congo (DRC) and the Republic of Côte d’Ivoire, with the aim of contributing to better management of retinoblastoma in this region. The specific objectives of this study were to describe the clinical presentation of retinoblastoma and the access to diagnosis and treatment in the Republic of Côte d’Ivoire and DRC. We also assessed the cost of treatment and the new initiatives started by parents’ association groups.

## PATIENTS AND METHODS

The population included all patients diagnosed with retinoblastoma between January 1, 2013 and December 31, 2014 in the pediatric oncology units in DRC (Lubumbashi, Kinshasa) and Côte d’Ivoire (Abidjan) and in an ophthalmology unit in DRC (Bukavu). Two of these units are pediatric oncology pilot units of the Franco-African Pediatric Oncology Group (Lubumbashi and Abidjan).^12,25^ The units admit and treat children diagnosed with cancer younger than 18 years of age.

The diagnosis of retinoblastoma was made after an ophthalmologic examination. The fundoscopy was performed only in rare cases of intraocular retinoblastoma, often without general anesthesia for various reasons, including lack of facilities, lack of anesthetic, or no access to a theater. However, the fundoscopy was performed in all accessible eyes and systematically in the contralateral eye. The diagnosis was confirmed after histopathological examination of biopsies or surgical specimens (enucleated eye).

In 80% of patients, the full examination included a bone marrow aspiration and lumbar puncture for analysis of the CSF. Because conservative treatment was not available, histopathological examination was indicated in all patients after enucleation or biopsy, as well as cytologic examination of the bone marrow and CSF. However, these examinations were financially unaffordable for some parents.

If possible, ultrasound was performed to identify intraocular lesions and, because of the lack of magnetic resonance imaging, ocular and brain CT scan was performed to identify extraocular lesions. Retinoblastoma was considered as familial when the retinoblastoma diagnosis had been made in a member of the family (mainly a parent, or another first-degree relative such as brother, sister, cousin, uncle, or aunt).

These children were admitted to services for disease management and included in the study after written or oral informed consent was provided by the parents or caregivers, according to the usual recommendations of the local possibilities and to the guidelines of the Franco-African Pediatric Oncology Group. This study received approval from the Ethics Committee of the University of Lubumbashi and the University of the Republic of Côte d’Ivoire.

### Collection and Analysis of Data

Patients’ medical records were reviewed by completing a similar form in all centers. Parents (one or both) or caregivers were interviewed in all cases to collect additional information. The pediatrician or ophthalmologist in each unit was responsible for the completion of the study.

The main variables included and analyzed were: date of birth, sex, age at diagnosis, family history of retinoblastoma, laterality, place of diagnosis, date of diagnosis, form of retinoblastoma (intraocular or extraocular), first symptom and the date of its appearance, date of first health professional consultation and stage of the disease according to the International Retinoblastoma Staging System classification of retinoblastoma,^26^ outcome, treatment achieved, average cost of treatment, parents’ marital status, and level of education of the parents. The time to diagnosis was defined as the time that had elapsed between the date of the first symptom/sign of retinoblastoma and the date when the diagnosis was confirmed by the ophthalmologist/pathologist. The time to treatment was defined as the time that had elapsed between the date of diagnosis and date of the treatment initiation. Entry and data analysis were conducted in the Epi Info 7.1 and SAS 9.3 (SAS Institute, Cary, NC) software.

## RESULTS

### Frequency of Retinoblastoma

A total of 119 children were admitted for retinoblastoma in the four medical centers over a period of 2 years. The total number of children admitted with cancer was 529. Three patients were not included because their records were incomplete. The distribution per unit was the following: Lubumbashi had a total of 64 cases of childhood cancer (19 cases of retinoblastoma), Kinshasa had 28 cases of retinoblastoma out of a total of 105 cases of childhood cancer, Bukavu had 39 cases of retinoblastoma out 56 cases of childhood cancer, and Abidjan had 33 out of 304 cases of childhood cancer. Median age at diagnosis was 3 years for patients with unilateral disease and 2 years for patients with bilateral disease.

The sex ratio was 1.1 (60 boys and 56 girls). Ninety-eight patients out of 116 (84%) had unilateral disease, and 18 patients (16%) had bilateral disease. Seven patients (6%) had a familial history of retinoblastoma; details are listed in Table 1. In Lubumbashi in 2015 there were 28 new cases of childhood cancer reported, and, out of those, 14 were retinoblastoma.

**Table 1:**
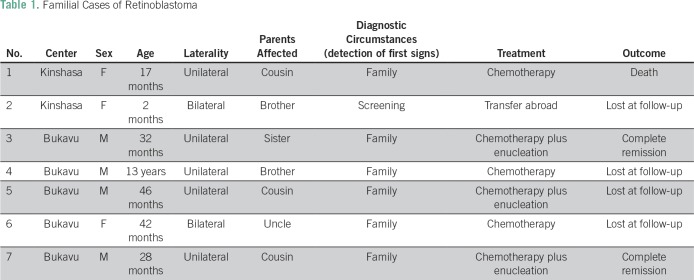
Familial Cases of Retinoblastoma

### Clinical Symptoms

Leukocoria was the first symptom noticed in most patients (82 children; 72%), followed by strabismus, found in 15 patients (13%). Three patients were discovered by screening. However, eighty-seven patients (76%) presented at the hospital sites with extraocular disease: orbital impairment (often exophthalmos or extraocular mass) and metastasis (invasion of the bone marrow, CSF, or brain).

The majority of patients (two thirds) were lost at follow-up. All patients treated received chemotherapy (the ophthalmologic center collaborates with a pediatric department of another hospital). The average cost of treatment (excluding chemotherapy offered by international associations, such as the Franco-African Pediatric Oncology Group [GFAOP]) was $1,354 (other details listed in Table 2). The first health care professional consulted was in most cases the nurse (34 cases), followed by the general practitioner in 27 cases, ophthalmologists in 24 cases, traditional healers in 13 cases, and pediatricians in two cases.

**Table 2:**
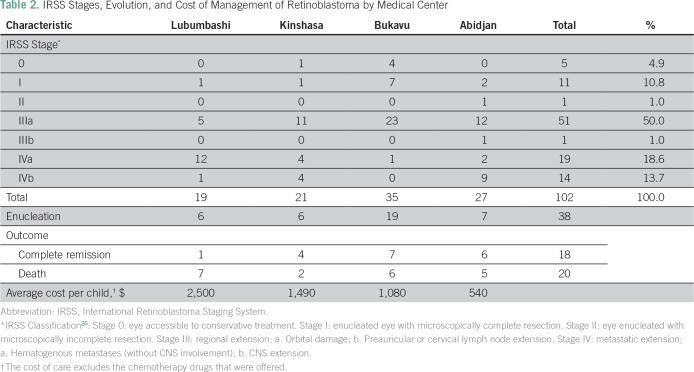
IRSS Stages, Evolution, and Cost of Management of Retinoblastoma by Medical Center

### Diagnosis

The median time to diagnosis was 8.5 months (range, 0.4 to 116.7 months; interquartile range, 3.3 to 27.6 months) and the median time to treatment was 31 days (range, 0 to 751 days; interquartile range, 10 to 73 days; Table 3). In most cases, the family noticed the first sign of retinoblastoma (94% of cases), followed by the general practitioner (5% of cases) and then by the nurse (1% of cases).

**Table 3:**
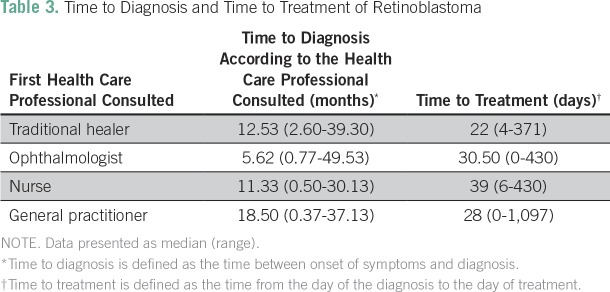
Time to Diagnosis and Time to Treatment of Retinoblastoma

The family decided to take the child to a referral center in 44% of the cases, whereas in 31%, the child was transferred by the referring physician. In most cases, the diagnosis was confirmed in an ophthalmology clinic or pediatric cancer GFAOP unit (77% of the cases). In 18% of cases, it was confirmed at another hospital and in 5% of cases at a health center. In 25% of cases, ultrasound and ocular and/or brain computed tomography scan were performed.

Our study found that the time to diagnosis was longer when the initial health care professional consulted was a general physician, compared with an ophthalmologist, and was also longer when parents were divorced, but neither of these differences was statistically significant. Also, no correlation could be established between the time to treatment, various forms of retinoblastoma, and age, gender, stage, health organization where the diagnosis was made, or parents’ educational level.

## DISCUSSION

This study confirmed that most cases of retinoblastoma in sub-Saharan African countries are diagnosed when extraocular extension is already present, as previously described.^13,17,19,27^ Similarly, delayed presentations and delayed treatment were observed. The mean time to diagnosis varied from 6.8 to 50 months, and the mean referral lag time was 1.7 months.^15-17,28^ Treatment of retinoblastoma may not be readily accessible in many low-income countries, because coverage by medical insurance is limited among this population. A study of cost of retinoblastoma management at Bamako (Mali) found the cost of 1,349.85 euros ($1,652.22). for extraocular forms.^29^ The cost was different between medical centers. Added to $600 of chemotherapy offered, we estimate the total cost at $1,954 per patient. Despite the donation of anticancer drugs, care was still financially unaffordable for many families.

In developed countries, and some middle-income countries, the median time to diagnosis varies from 0.5 months to 3.5 months.^11,20,21,30-32^ The median treatment lag time was 7 days (0 to 45 days) in a French series, and < 15 days for 118 of 121 patients.^33^

The DRC, a country of close to 70 million people, known as the country fighting Ebola and yellow fever in the past, has a total 4.3% of gross domestic product (GDP) spent on health^34^ of the total of $400/GDP per capita.^35^ Children younger than 15 years represent close to half of the population (44.4%).^36^

Ivory Coast, known as the world’s largest producer of cocoa, has a population of approximately 22 million, where children younger than 15 years of age represent close to 40% of the total population.^37^ The country had a GDP per capita of $1,491.66 in 2015,^38^ an expenditure on health per capita of $187, and the total expenditure on health as percentage of GDP of 5.7%.^39^

Thus, we were limited in our study by the precarious conditions that have limited diagnostic examinations and assessments: fundoscopy often performed without general anesthesia and without retinal camera, orbit and full-brain imaging not performed in most cases, and insufficient experience of our investigative services and care (pediatrics, ophthalmology, and pathology). Thus, we were unable to describe the international classification of the rare intraocular retinoblastoma cases in this study. However, the International Retinoblastoma Staging System classification could be used. The financial inaccessibility of treatment as well as belief, delayed diagnosis, and lack of psychosocial support could explain the high proportion of people lost at follow-up and the low rate of complete remission. Other studies are needed to investigate the compliance factors.

The responsibility of delayed diagnosis and management must be shared among all health care professionals, whether modern or traditional. Traditional healers are far from being the only ones to be responsible for this delay in diagnosis.^40^ In sub-Saharan Africa, a high proportion of parents have minimal education,^14^ and the lack of awareness of retinoblastoma remains predominant in all communities (public and health care professionals).^5,13,14,28^ However, to our knowledge, the relationship between the educational level of parents and the lag time of diagnosis and management of retinoblastoma has not been proven. In our study, the diagnosis delay is longer for divorced families than married partners, which we attribute to the number of children and increased responsibility of the mother and in most instances of the grandmother—but the difference is not statistically significant. Other studies have shown a relationship between poor socioeconomic conditions (which could be linked to educational level) and advanced stages of retinoblastoma.^41^

The median age at diagnosis of retinoblastoma in our study was 3 years for unilateral cases and 2 years for bilateral cases, confirming the late diagnosis in sub-Saharan Africa. As the children present with advanced disease, the clinical presentation can sometimes be dramatic. Bilateral forms were observed in 16% of patients (18 of 116); it is recognized that 40% of patients have bilateral retinoblastoma, according to international data.^3^ Sub-Saharan Africa studies have found 11% to 33% of patients with retinoblastoma to have bilateral disease.^13,14,24,27,28^ Because our statistics are based on hospital data, no reliable conclusion can be drawn. There is often a predominance of males in retinoblastoma,^10,11,14,23,24,28,31,40^ but this was not observed in our case.

In 6% of patients, there was a family history of retinoblastoma. Only one of these patients was among the three children whose retinoblastoma was discovered by screening. Most of the affected parents were siblings or cousins, ​​and there was one young uncle. The survival of retinoblastoma in our environment would mainly concern recent cases; this would justify the absence of a direct ascendant affected (father or mother). These familial forms were undoubtedly low-penetrance forms, because we observed mainly children of unaffected parents.^42^ In our countries, where testing for the RB1 mutation is not yet possible, these factors, as well as the bilateral or multifocal character, suggest a genetic predisposition and can help select at-risk children. More attention should be given to ophthalmologic monitoring for screening of the disease.^3^

Retinoblastoma is easy to diagnose, and results are excellent if treatment is started early. Unfortunately, for us it remains a challenge, because it continues to be diagnosed late in our African countries; this results in children losing not only their eyes and vision but also, in many cases, their lives. All health care professionals, as well as our traditional healers, should join forces to increase awareness in the general communities. Parent groups as well as international organizations will remain vital in the future as more children are diagnosed and need better and efficient treatment support.
